# Emotional Competence of Early Childhood Educators and Child Socio-Emotional Wellbeing

**DOI:** 10.3390/ijerph18147633

**Published:** 2021-07-18

**Authors:** Angelica Arace, Laura Elvira Prino, Donatella Scarzello

**Affiliations:** Department of Philosophy and Education Sciences, University of Turin, 1024 Turin, Italy; lauraelvira.prino@unito.it (L.E.P.); donatella.scarzello@unito.it (D.S.)

**Keywords:** early childhood educators, emotional competence, socio-emotional wellbeing, child-educator attachment, ECEC quality

## Abstract

Background: Early childhood educators are attachment figures for babies and play an important role in emotion socialization. This study aims to analyze the role of educators as emotional socializers and its relationship with infants’ social competence and attachment security, considering various characteristics of educators (age, years of experience, level of knowledge of development and parenting) and the context (day-care center–family communication). Methods: 563 infants attending day-care centers (age: M = 25.98 months SD = 5.41) and their 223 early childhood educators (age: M = 42.61 SD = 11.02) took part in this study. The educators completed: CEESQ—Crèche Educator Emotional Style Questionnaire, Information Sources Questionnaire, two sub-scales of KIDI—Knowledge of Infant Development Inventory, QRS-F—Questionnaire on the Relationship between Services and Families, QPI—Questionnaire on Peer Interactions, and AQS—Attachment-Q-Sort. Results: Results showed that the educator’s coaching style has a relationship with attachment security and social skills and is positively correlated with the educators’ emotional self-efficacy and with the level of communication between day-care centers and families, while the correlation with knowledge of parenting is weak. Conclusions: These findings highlight the importance of enhancing not only educators’ knowledge about educative strategies, but above all their emotional competence to promote children adaptation to day-care centers.

## 1. Introduction

Childhood is a particularly important period of development in terms of forming emotional relationships that will have a long-term impact on children’s socio-emotional development and psychological well-being; indeed, one important developmental task in the early years is precisely forming an attachment with caregivers, whose role is an important one in ensuring the child’s emotional adjustment, well-being, and social skills [1,2]. Earlier research has analyzed the relationship with mothers as primary attachment figures; however, awareness has progressively grown that children establish more than one attachment relationship during their infancy; the perspective of multiple attachments [3] has therefore led to an increasing focus on the relationships that children establish with other family caregivers, the father first and foremost, but also professional caregivers such as nursery educators and preschool teachers, to whose care an ever greater number of children are entrusted on a daily basis.

### 1.1. The Quality of Professional Caregiving

The perspective of multiple attachments has given rise to an extensive debate as to the quality of the educational experience and non-family care and the effects of attending day-care centers on child psychological development, with research results that are not always in agreement. Some studies have in fact emphasized positive fallout in terms of cognitive, linguistic and social development in children [4,5], while others have instead reported an increase of externalizing problems and stress levels [6,7]. Several variables, in fact, can mediate the effects of attending day-care centers, leading to different development results and these range from the child’s characteristics (such as age, gender, temperament, socio-emotional competence), those of the family (members, social-economic and cultural status, quality of parenting and of attachment relationship), the day-care center (quality standards offered, quality of the relationship established between educators and children, number of peers in the group, child–educator ratio) and the social-cultural variables of the context.

What is agreed upon is that ECEC quality can also have a long-term impact on children’s development and well-being [8,9] and that this quality concerns both structural variables (like the number of peers in the group and the ratio of adults to children) and process variables, which are instead related to the quality of relational exchanges [10]. Educators offer a more supportive, sensitive quality of caregiving when there are favorable structural variables, such as being able to work in small groups [10,11,12,13]; however, a significant quantity of research suggests that it is not only a high-quality organization that facilitates the development of good relationships, but rather that the quality of the child-educator relationship is itself a key aspect of the quality of the educational service [14,15,16,17,18,19,20]. An in-depth analysis of the impact of the relationship with a professional caregiver on infant development is therefore essential, in order to offer all children a solid start in life, through quality care and education.

### 1.2. The Current Study

Given that numerous studies have shown that a parent who promotes secure attachment is a parent who is also able to tune in carefully to the child’s emotions and foster his/her regulation [21,22,23], in this study, we chose to analyze the role played by the emotional competence of the educator in promoting the socio-emotional security and well-being of the child at the day-care center; given that the child’s socio-emotional competences start developing right from early childhood, and that the family, although the first, is not the only context of emotional socialization, it is in fact important to also investigate the role of educators and teachers in emotional socialization of pre-school children and the effects in terms of attachment security [24,25,26,27,28].

Meta-emotion philosophy [25,26] is the reference model; it is a construct that identifies the combination of experience and beliefs in the adult with respect to his/her emotions and those of the child. Traditionally it has been investigated mainly in regard to parental behavior, neglecting to consider other caregivers, such as early childhood educators or preschool teachers, who can be equally significant emotional socializers during infancy [27]. As shown in the literature, educators can react to children’s emotions showing empathy and offering comfort, or by seeking to distract children from the emotion experienced, or perhaps helping them to find their own coping strategies to deal with emotions; alternatively, by contrast, they may ignore children’s emotions and show a reaction of refusal [29,30]. The term ‘meta-emotion philosophy’ is used to refer specifically to the adult’s perception of the most appropriate manner to promote children’s emotions expression and regulation, as well as the level of knowledge, acceptance and regulation of their own emotions [26]. The choice to investigate meta-emotion philosophy is supported by the fact that recent research has shown how the ‘emotional work’ of educators has a significant impact on the quality of interaction between educators and children, as well as between educators and parents and within the group of educators [31,32].

More specifically, the study has the following objectives:

- To verify that the style of emotion socialization adopted by the educator has a relationship with children’s social skills and attachment security; we can hypothesize, in fact, in line with what is extensively acknowledged in studies of parenting, that a greater capacity on the part of the educator to accept and regulate a child’s emotions is associated with a higher level of social competence and security in the child. Any gender differences will also be analyzed.
- To analyze whether the emotional competence of the educator with respect to handling their own emotions impacts the emotional socialization style adopted in regard to children; we expect to see that an educator who is able to recognize and regulate children’s emotions will also show greater awareness of their own emotions and a greater capacity to regulate them. The influence of personal variables of the educator, such as age, level of education, years of service, having/not having at least one child of their own, knowledge of child development and functional parenting, will also be analyzed. In general, research has shown a positive association between professional training, quality of day-care and children’s outcomes [33,34]. Years of work experience can also increase educators’ sense of self-efficacy and make them more skilled in both handling their own emotions and those of the children [35].
- To analyze whether the style of emotional socialization is associated with contextual variables and, in particular, the quality of communication between educators and parents, as perceived by the educator. We hypothesize that the emotional competence of the educator should facilitate not only the development of the relationship with the child, but also that with the families and that educators with better emotional skills will show a greater engagement with the families.

## 2. Materials and Methods

### 2.1. Sample

Participants were contacted after approval of the research protocol by the university ethics committee.

For this study, 40 day-care centers in a large city in Northern Italy were invited to participate: they were selected from a larger basin of 55 day-care centers divided into 8 districts with different demographic and socio-cultural characteristics, randomly choosing 5 day-care centers for each district in order to guarantee the territorial representativeness of the analysis sample.

Participants were 223 educators, almost all women (97%) and Italian (99%), aged between 23 and 65 years old (M = 42.61; SD = 11.02), in most cases (68.7%) in possession of a high school diploma (29.5% have a university degree), with a very varied length of service (range: 4 months to 41 years; M = 17.53; SD = 11.35). Most (68.3%) were in a stable relationship with a partner: married (47.5%) or cohabiting (20.8%). A total of 58.7% had children.

Educators completed questionnaires for 563 children (56% males and 44% females), aged between 12 and 37 months (M = 25.98; SD = 5.41). The children’s socioeconomic status (SES), measured using Hollingshead index [36] to measure parental occupation and educational level, was medium-high (M = 46.38; SD = 12.41).

Table 1 summarizes the characteristics of the children, subdivided by gender; the preliminary analysis of the sample shows that there are no significant differences between males and females in age, age of entry into day-care and daily hours spent at the day-care center.

### 2.2. Measures

#### 2.2.1. The CEESQ

The emotional socializer task of the educator was assessed through the CEESQ (Crèche Educator Emotional Style Questionnaire; [25,26]). The CEESQ is a self-report questionnaire with two sections; each item is scored on a 5-point Likert scale ranging from 1 (rarely or never) to 5 (very often). The first section refers to the capacity of the educator to respond to children’s emotions and support them in emotional regulation and it has 3 sub-scales:

- Coaching, comprising 7 items (awareness, acceptance and regulation of the children’s emotions. Example: “When a child is angry, it is an opportunity for getting close”)
- Dismissing, comprising 5 items (little awareness of the children’s emotions, idea that negative emotions are inappropriate and tendency to ignore them. Example: “The children will learn to manage their emotions by themselves”)
- Self-efficacy as emotional socializer, comprising 6 items (assessment of oneself as being capable of differentiating the emotional status of the children and helping them understand and manage emotions. Example: “I can get the children to express all of their emotions”).

The reliability coefficients for the three sub-scales are α = 0.69, α = 0.63, and α = 0.81, respectively, and are sufficiently in line with the psychometric characteristics reported by the authors (α = 0.75, α = 0.78, and α = 0.70, respectively).

The second section refers to the emotional competence of the educator and consists of two sub-scales:

- Personal emotional self-efficacy of the educator, comprising 10 items (awareness and ability to manage one’s own emotions. Example: “I am able to express what I feel”).
- Denial of emotions, comprising 4 items (failure to accept one’s own emotions. Example: “I perceive my negative emotions as something to defend myself against”).

The reliability coefficients of these two sub-scales are α = 0.82 and α = 0.57, respectively, in line with the Italian instrument validation data (α = 0.86 and α = 0.59, respectively).

#### 2.2.2. The KIDI and the Information Sources Questionnaire

To assess the educators’ knowledge of the development of young children and parenting, the Italian version [37] of the KIDI (Knowledge of Infant Development Inventory; [38]), was used. Subjects indicate the degree to which they agree or disagree with each of the statements, or opt for ‘not sure’. The scoring allocates 1 point for each correct answer, −1 for each incorrect answer and 0 points for each ‘not sure’. Two sub-scales were administered in this study:

- Principles (17 items): contains affirmations, axioms and clichés, regarding development processes. Items regarding the description of general abilities as well as typical and atypical development were also included under this section (e.g., “Babies only understand words they can say”).
- Parenting (14 items): these items concern instrumental beliefs about parenting strategies, infant management and responsibilities of being a parent (e.g., “Fathers are naturally clumsy when it comes to taking care of babies”).

The KIDI has been determined to have an internal consistency that ranges from 0.50 for professional caregivers to 0.82 for parents and the test-retest reliability coefficient ranges 0.80–0.92 [38]. The KIDI is comparable with other measures of caregiver knowledge of child development thereby demonstrating construct validity [39]. In our study, the reliability coefficients of the two sub-scales are α = 0.58.

The “Information Sources Questionnaire”, prepared specifically starting from the catalogue of previous experience with infants [38], was used to investigate the sources of information applied by educators to gain knowledge about children. Educators were asked to indicate, on a 5-point Likert scale, ranging from ‘nothing’ to ‘very much’, what they had learnt about children from a series of 7 sources: family (parents, siblings, grandparents); friends or other adults with children; their partner; mass media (radio, television, films); doctors, nurses, midwives; teachers/educators; the reading of articles or books. The reliability coefficient of the scale is α = 0.69.

#### 2.2.3. The QPI

The social skills of the children at the day-care were assessed using the QPI (Questionnaire on Peer Interactions; [40,41]), validated in the Italian context. It consists of 22 items that assess the ability to interact with peers. Educators indicate the frequency of each behavior according to a 4-point Likert scale (1. ‘rarely’ to 4. ‘very often’). The QPI assigns scores on four scales: 1. Negative social behaviors, 7 items (example: “physically attacks the other children”); 2. Positive social behaviors, 5 items (example: “if a playmate is crying for any reason, he/she approaches him/her and tries to console him/her”); 3. Difficulties in social participation, 5 items (example: “he/she needs to be solicited by the educator in order to play with the others”); 4. Popularity, 5 items (example: “the other children spontaneously seek to involve him/her in playing together”). By re-encoding the items that indicate negative behaviors (so that high values indicate a lower occurrence), we have also identified a global index of social skills, given by the sum of the single scores [42]. The reliability coefficients of the scales are: Negative social behaviors: α = 0.57; Positive social behaviors: α = 0.77; Difficulties in social participation: α = 0.63; Popularity: α = 0.83; Global index: α = 0.77.

#### 2.2.4. The AQS

To assess the quality of the emotional bond between educator and child, a questionnaire was used drawn from the AQS (Attachment Q-sort; [43]) and proposed by Cassibba and D’Odorico [44]. The questionnaire comprises 29 items that allow for the observation and recording of child’s secure behaviors toward educator at the day-care center. Each item describes a typical behavior evidencing a secure relationship between the child and the educator (e.g., “If the child is angry or hurt, he/she prefers to be consoled by his/her reference educator rather than by other adults”) and the educator indicates, on a 5-point Likert scale, the degree of similarity with the behavior of the observed child (from ‘very different’ to ‘very similar’). The total score was calculated, given by the sum of the answers given to each item. Higher scores suggest attachment relationships characterized by greater security with the educator. The reliability coefficient of the scale is α = 0.89.

#### 2.2.5. The QRS-F

To assess the relationship and communication between day-care center and children’s families, the specifically constructed “Questionnaire on The Relationship Between Services and Families” (QRS-F) was used. It comprises 15 items identified on the basis of previous literature on the topic [45] (for example: “Meetings are organized with families to provide an opportunity for listening and comparing ideas on educational matters”) and subjects indicate the degree to which they agree with them, on a 5-point Likert scale (from ‘not at all’ to ‘very much’). The reliability coefficient of the scale is α = 0.79.

### 2.3. Data Analisys

Data analysis was performed by means of the statistical package SPSS, version 26.

First and foremost, descriptive analyses were prepared for all the variables considered and the sample of children was checked for gender differences, using the *t* test analysis for independent samples. The second step was to explore the presence of associations between the emotional socialization style of the educators (measured through the first section of the CEESQ), the social skills of the children at the day-care center (measuring using the QPI) and the security level of their attachment with the educator (measured using the AQS). To this end, bivariate correlation analyses were performed, and thereafter, two multiple linear regressions were performed using the stepwise method, separately for males and females; independent variables: coaching style, dismissing style and self-efficacy as emotional socializer; dependent variables: total social skills score (regression 1) and security score (regression 2).

Through bivariate correlation analysis and variance analysis, depending on the type of variables considered, we analyzed the association between the style of emotional socialization of the educators (first section of the CEESQ) and personal variables (age, level of education, parenthood status, years of experience as educators, knowledge of development and parenting—measured through the KIDI—reference sources for acquiring knowledge, personal emotional competence—measured through the second section of the CEESQ) and contextual variables (perceived quality of communication between nursery and family—measured using the QRS-F).

## 3. Results

### 3.1. Style of Socialization and Emotional Competence of Educators: The Results of the CEESQ

The descriptive analysis of the scores recorded in the first section of the CEESQ, which examines the educators’ response to the children’s emotions, shows that the professionals of our sample tend to use a coaching style for preference, rather than a dismissing style and that self-efficacy as emotional socializer is medium-high (Table 2). The results are in line with recent studies carried out on similar samples across Italy and with the instrument validation data [24,26,28].

An analysis of each item was also carried out, aimed at identifying those that obtained a higher mean score (>4) and those that obtained a lower score (<2). In the first section of the questionnaire, the two statements on which the educators are most agree (mean score >4) are: Item “Children’s sadness is an emotion worth exploring” (range: 1–5; M = 4.16, SD = 0.76) and item: “The contribution of early childhood teachers to the emotional development of young children is fundamental at the day-care center” (range: 1–5; M = 4.03; SD = 0.78), while in just one item a score of below 1 was recorded: “If children are sad, I don’t get involved unless it lasts too long or it is too intense” (range 1–5; M = 1.68; SD = 0.92).

Most educators are aware of their role in promoting children emotional development and believe it to be important to explore and tune into negative emotions, like sadness. Our data, however, show that not all emotions can be managed as easily by the educators; sadness is the emotion that the educators ‘handle’ with least concern, while anger is the most critical emotion (Table 3). Indeed, only a small minority of professionals declare that they do not become involved with children’s sadness (very often/often = 5%) and almost all the sample believe that sadness is an emotion worth exploring (very often/often = 84%), while it is less frequent that educators consider child’s anger as an opportunity to support him/her (very often/often = 59.5%) and feel able to support an angry child (very often/often = 59%).

As indicated by the values of the Emotional self-efficacy and Denial of emotions scales (Table 4), the analysis of the second part of the CEESQ on educators’ personal emotional competence shows that as a whole, educators feel able to manage their own emotions, which they do not believe to be a hindrance in their interpersonal relationships. In greater detail, educators declare that they have a good awareness of their emotions (range: 1–5; M = 3.76; SD = 0.75) and their causes (range: 1–5; M = 3.60; SD = 0.73) and that they feel able to regulate even more intensely activated emotions, such as anger (range: 1–5; M = 3.63; SD = 0.69) and fear (range: 1–5; M = 3.42; SD = 0.70). In addition, they feel able to adequately express and communicate what they feel (range: 1–5; M = 3.47; SD = 0.75) and do not agree with the idea that negative emotions are something against which they should defend themselves (range: 1–5; M = 1.87; SD = 0.83). In accordance with the instrument validation data, the personal emotional self-efficacy scale negatively correlates with the denial of emotions scale (r = −0.32; *p* < 0.01) and both scales have average scores in line with that reported by the authors.

The comparison between the mean scores and additional correlation analyses has not revealed any significant differences with respect to the level of education of educators, age, and years of service in the day-care and having/not having at least one child of their own, neither for the first part of the CEESQ (with respect to which additional analyses will be given in par. 3.6), nor indeed for the second part of the CEESQ.

### 3.2. Educators’ Knowledge of Child Development and Functional Parenting Strategies: The Results of the KIDI and the Information Sources Questionnaire

The results that emerged from the administration of the KIDI indicate that educators have good knowledge of the principles and stages in child development and the most functional parenting strategies (Table 5), without any differences worthy of note in terms of age, level of education, years of professional experience and whether or not they have their own children. In line with the instrument standardization data, the two scales show a positive correlation (r = 0.30; *p* > 0.01). The error rate for each item is in line with the instrument’s normative data; overall, the higher error percentages relate to items that describe a deterministic vision of development (e.g., “The way the parent responds to the baby in the first few months of life determines whether the child will grow up to be happy and well-adjusted, or moody and a misfit”, or “The baby’s personality (individuality) is set by 6 months of age”), and highlight a difficulty in recognizing the influence of individual differences in behavior (e.g., “If a baby is shy or fussy in new situations, it usually means that there is an emotional problem”).

As regards the sources of information used by educators to increase their knowledge, expert sources prevail, such as reading books and articles, as well as discussions with other colleagues; informal sources, such as friends and family and mass media are instead less relevant (Table 5). Educators with a higher level of education make more use of expert sources (F_(4, 209)_ = 3.04; *p* < 0.01); vice versa, educators with at least one child of their own declare that their knowledge of child development and parenting comes from their family network (F_(1, 210)_ = 8.56; *p* < 0.01), discussions with doctors (F_(1, 210)_ = 9.23; *p* < 0.01) and, above all, their partner (F_(1, 205)_ = 24.34; *p* < 0.001).

### 3.3. Children’s Social Skills and Attachment Security with the Educator: The Results of QPI and AQS

As regards children’s attachment security with the educator and their social skills, a descriptive analysis of considered variables was first carried out, separately for boys and girls.

As concerns attachment with the educator, the results show that boys have a lower security score than girls and that is confirmed by the t test for independent samples. Boys also show lesser social skills than girls, as has already been pointed out by previous studies [46]; the t test for independent samples shows a significantly lower level in the global QPI score and on the positive social behavior scale and a significantly higher score in terms of difficulty in social participation and negative social behavior. Only the scale of popularity does not show gender differences (Table 6).

The analysis of each item confirms the results of previous research [46]; the greatest differences between males and females (*p* ≤ 0.01) are found in specific behaviors, some more typically seen in girls (“If a schoolmate cries for any reason, the child gets close and tries to console him”; “If a schoolmate is in trouble, the child tries to help”; “The child manages to play collaboratively with a schoolmate”) and others more frequently observed in boys (“The child is the object of physical aggression by other children”; “The child has to be stimulated by the educator/teacher before he plays with others”). Therefore, data show that females are more empathic and more likely to engage in pro-social behavior, while males are more frequently involved in conflict and more prone to social withdrawal.

The correlation analysis shows that the children who exhibit mainly positive social behaviors are also the most popular, while the children who exhibit mainly negative social behaviors are those who experience greatest difficulty in social participation, with no difference between males and females (Table 7).

Security of attachment and social competence correlate positively; the children with the highest security scores are assessed by the educator as more competent in peer relations and more popular, while children with a more insecure attachment are described as more likely to implement negative social behaviors. In this case, too, the correlation analysis does not show any gender difference in the children (Table 7).

Finally, for both males and females, children’s age correlates positively with social skills; the increase in age is associated with an increase in positive social behaviors and in popularity and with a decrease in negative social behavior as well as difficulties in social participation. Instead, there is no association between age and attachment with the educator (Table 8).

### 3.4. The Quality of Communication between Educators and Family: The Results of the QRS-F

Overall, the level of communication between educators and parents is described as good and satisfactory (range: 19–71; min = 28; max = 69; M = 48.88; SD = 7.72; Skewness = 0.23; Kurtosis = −0.04). In particular, everyday communications are described as constant and profitable when children arrive in the morning and leave in the afternoon (range: 1–5; M = 4.05; SD = 0.783); in addition, educators declare that they inform parents regularly about their children’s progress (range: 1–5; M = 3.86; SD = 0.77). The poorest aspects in day-care–family communication instead relate to the active participation of parents in planning (range: 1–5; M = 2.75; SD = 1.14) and evaluating the educational activity (range: 1–5; M = 2.75; SD = 1.04), as well as the space organization to welcome parents (range; 1–5; M = 1.97; SD = 0.97).

### 3.5. Children’s Social Skills and Attachment Security with the Educator: The Role of Educator Emotional Socialization Style

To analyze the hypothesis of an association between social skills and attachment security with the educator and the emotional socialization style adopted by the educator, we first explored the existence of correlations between the variables.

Social skills correlate positively with the coaching style (global score: r = 0.172; *p* < 0.000; positive social behaviors: r = 0.11; *p* = 0.009; popularity: r = 0.20; *p* < 0.000; negative social behaviors: r = −0.19; *p* < 0.000) and negatively with the dismissing style (global score: r = −0.14; *p* = 0.003; positive social behaviors: r = −0.14; *p* = 0.001; popularity: r = −0.19; *p* < 0.000), while no significant associations are revealed with self-efficacy as emotional socializer.

The emotional socializer style is also associated with attachment security with the educator; more specifically, security of attachment correlates positively with the coaching style (r = 0.23; *p* < 0.000) and with self-efficacy as emotional socializer (r = 0.15; *p* = 0.001) but does not correlate with the dismissing style.

To verify that emotional socialization style is associated with children’s social skills and attachment security with the educator, two regression analyses were performed using stepwise method, entering coaching style, dismissing style and self-efficacy as emotional socializer as independent variables. In the first analysis, the total score on social skills was entered as dependent variable, and in the second analysis, the attachment security score. To highlight any differences in gender, the analyses were carried out separately for males and for females (Table 9).

The results suggest that for males, coaching style is positively associated with social skills, while dismissing style is negatively associated. Instead, in females, social skills are not associated with the educator’s emotional socialization style.

The educator’s coaching style instead has a significant relationship with attachment security score for both boys and girls.

### 3.6. Educator’s Emotional Socialization Style: Personal and Contextual Variables

To verify the hypothesis that an emotional socialization style connects to personal variables of the educator, a correlation analysis was performed between the emotional socialization styles (coaching, dismissing and self-efficacy as emotional socializer) and the following variables: personal emotional competence of the educator (personal emotional self-efficacy and denial of emotions), age, level of education, years of experience as an educator, knowledge acquired on child development. Instead, in order to investigate the relationship with contextual variables, a correlation analysis was performed between the emotional socialization styles (coaching, dismissing and self-efficacy as emotional socializer) and the quality of educator-family communication. The results are summarized in Table 10.

The correlation analysis shows an association between emotional socialization style in regard to children and the educator’s personal emotional competence; the coaching style positively correlates with the emotional self-efficacy, while the dismissing style correlates with the denial of emotions; self-efficacy as emotional socializer correlates positively with personal emotional self-efficacy and negatively with the denial of emotions.

The results of the analyses also indicate that the emotional socialization style adopted in regard to children is instead not associated with the age of educators, nor their level of education, years of experience or whether or not they are parents, as highlighted in this latter case by the variance analysis (Coaching: F_(1, 206)_ = 0.000; *p* = 0.998; Dismissing: F_(1, 198)_ = 0.005; *p* = 0.944; Self-efficacy as emotional socializer: F_(1, 206)_ = 1.208; *p* = 0.206).

In addition, the association between emotional socialization styles (coaching, dismissing and self-efficacy as emotional socializer) and knowledge of development and parenting, measured through the KIDI, and reference sources for the acquisition of this knowledge were analyzed. The correlation analysis between knowledge of the educator measured by the KIDI and emotional socialization style highlights that knowledge of parenting is positively associated, albeit only weakly, with the coaching style and negatively with the dismissing style. A weak negative association between dismissing style and knowledge of development principles is also revealed.

Finally, in order to verify the hypothesis that the emotional socialization style is connected with variables relative to the day-care context, a correlation analysis was performed between emotional socialization style (coaching, dismissing and self-efficacy as emotional socializer) and quality of communication with families, measured by means of the QRS-F. More specifically, day-care–family communication correlates positively with both coaching style and self-efficacy as emotional socializer (Table 9).

## 4. Discussion

### 4.1. Educator’s Role as an Emotional Socializer: Personal and Professional Variables

The main aim of this research was to investigate the educator’s role as an emotional socializer and its influence on the quality of children’s attachment relationship and their social skills at the day-care center.

In line with previous research on the meta-emotion philosophy [21,26], educators describe their emotional socialization style mainly according to the characteristics of the coaching style; they believe that they are able to understand the child’s emotions and offer adequate support in emotional regulation processes, thereby perceiving good levels of self-efficacy as emotional socializers. Most educators are in any case aware of their role in promoting the emotional development of the child and believe it to be important to tune into their emotions, both positive and negative. Not all negative emotions can, however, be managed with the same effectiveness by educators; indeed, they declare that they can better ‘handle’ sadness in children but are instead in greater difficulty with anger management. This latter is, in fact, linked to externalizing type behaviors that can be more difficult to regulate in a group context, where the educator is called to meet the needs and emotional expressions of multiple children at the same time.

Results also confirm the hypothesis that educators able to respond appropriately to children’s emotions are adults able to effectively manage their own emotions too, showing good levels of personal emotional self-efficacy, as also shown by other research [26]. By contrast, educators who take on a dismissing style in regard to children’s emotions, characterized by disengagement or refusal, obtain higher scores on the scale of denial of one’s own emotions, experienced as an obstacle that is difficult to overcome. Educators aware of their emotions and inclined to accept them and regulate them effectively are therefore able to act as emotional ‘coaches’ towards children, activating modeling and scaffolding processes when faced with emotionally activating situations, while educators with lesser personal emotional competence find it more difficult to empathize with children’s emotions and are unable to support them in developing their emotional competence. Nevertheless, infants have greater need for caregivers able to act as external emotion regulators.

Professional training, years of experience, and knowledge acquired about child development would not appear to have a significant association with the emotional competence of educators, nor indeed with respect to how they manage their own emotions or those of the children. No statistically significant correlations were found between level of education and years of service on the one hand, and style of socialization and emotional self-efficacy on the other. Only weak associations were found with knowledge of the principles of child development. This result can be interpreted on the basis of the fact that, as a rule, entry and in-service training of educators mainly regards theoretical and methodological types of knowledge and only more rarely does it also cover reflective training on the emotional aspects of educational work. A good level of theoretical knowledge about child development and the most functional educational strategies, also obtained from expert sources such as scientific articles or books, as in the case of our sample, does not, therefore, suffice to promote the competence and emotional self-efficacy of educators. It is probable that personal characteristics, such as sensitivity and emotional availability, are more important variables than training and education in explaining the educator’s capacity to build a satisfactory emotional relationship with the child, as also supported by other studies [14,47].

### 4.2. Educator’s Role as an Emotional Socializer: Effects on Children’s Attachment and Social Competence

As regards the influence of the emotional socializer role of educators on the quality of children’s attachment relationship and on their social skills at day-care center, the initial hypotheses were confirmed.

In line with what is extensively known with respect to the positive relationship between the emotional competence of the family caregivers and the child’s development, the emotional socialization coaching style of the educator is positively associated with the security of attachment, both for males and females; an adult who embraces, contains and regulates the child’s emotions constitutes a secure basis on which to rely. The fact that professional caregivers are important attachment figures for children attending day-care centers is now known within the theoretical perspective of multiple attachments [4,48,49]. Some studies even show a compensatory role played by a secure attachment relationship with the educator, which can partially reduce the negative effects on the social skills of a child brought about by an insecure attachment relationship with the maternal caregiver [50]. The same Richard Bowlby stresses that babies and toddlers in non-parental daycare can avoid stress and anxiety if they develop a lasting secondary attachment with one caregiver who is consistently accessible to them [51]. Our research, however, revealed gender differences in the possibility of constructing a secure attachment relationship with the educator; males, in fact, show lower security scores than females. Gender differences observed in the AQS scores are similar to that obtained by Howes and Smith [49] and Commodari [52] in studies on attachment to non-parental figures. In accordance with that declared by Crockenberg [53] and Meland et al. [54], it is likely that boys in preschool age have greater need for a well-structured care environment with a highly sensitive, responsive caregiver than girls, due to a greater immaturity in their socio-emotional development; in general, research suggests that boys would appear to be more susceptible to quality levels of childcare in educational contexts, having shown, for example, that low-quality child care is strongly associated with the presence of behavioral problems in males, while the same association is weak or non-existent for females [55,56].

Overall, children with a secure attachment with the educator show greater pro-social behaviors in response to peers’ emotions and display mainly positive social behaviors. In contrast, children with higher levels of insecurity mainly show negative externalizing social behaviors and greater difficulty in social participation, withdrawing and isolating themselves. These results are in agreement with other research on the positive impact on development of children and their adjustment at day-care center, which has highlighted how a secure attachment promotes a greater capacity for empathy in children, an ability to engage in cooperative relationships with peers and a mastery of the environment [4,48,49]. In addition, more secure children often feel more confident in the caring environment, have enhanced cognitive activity, and will be more successful at learning [57,58,59].

It can therefore be declared that the emotional competence of the professional caregiver makes it more likely that the child will develop a secure attachment relationship, promoting social skills in a cascade fashion. A direct link between the coaching style of the educator and the social skills of the child cannot; however, be excluded. In this regard, although the results of our research show similar effects on attachment security in both males and females, they instead highlight gender differentiated effects on social skills; the coaching style of the educator has, in fact, a positive relationship with social skills in boys, but not in girls; in the same way, the dismissing style has a negative relationship with the social skills of boys only. It is, therefore, likely that the direct effect of the emotional socialization style of the educator on the social skills expressed at the day-care center differs by gender; in light of previous research [60], it can be hypothesized that girls, who generally have greater socio-emotional competence in peer relationships ahead of their male counterparts, need less support from educators. Several studies in fact sustain that in preschool age, boys have greater difficulty in autonomously regulating their emotions, lesser skill in regulating negative levels of arousal and greater sensitivity to stress coupled with lesser abilities in perspective taking, all aspects that could reduce their capacity for adjustment in group contexts and make them more susceptible to the action of professional caregiver as external regulator of their emotions [46,53,56,61,62,63]. The early socio-emotional competence developed by girls in preschool age could instead be the consequence of gender-differentiated parental practices; parents more easily involve their daughters in emotional dialogues and better strengthen empathetic and pro-social behaviors [60,64,65]. In all, therefore, our study provides further confirmation that females appear to have an advantage over males in various domains of development which are especially important in a day-care context; females show a greater number of positive social behaviors and fewer negative social behaviors, as well as lesser difficulty in social participation. They are also more empathetic and engage more easily in pro-social behavior, while males are more frequently involved in conflict with peers and more likely to withdraw socially. Overall, females are better suited to face the development tasks typical of preschool educational contexts, appearing to be more independent, better at regulating themselves and communicating [54,66,67].

### 4.3. Educator’s Role as an Emotional Socializer: Effects on the Relationship with Families

Finally, the last objective of the study was to explore the relationship between the educator’s emotional competence and the quality of communication with parents, assuming that the educator’s emotional competence facilitates not only the construction of a good relationship with the child, but also with his/her family. In the presence of a coaching emotional socialization style and good levels of personal emotional self-efficacy and self-efficacy as emotional socializer, educators consider communication with parents to be an important aspect of their work, which contributes towards their job satisfaction. Other studies have also revealed that positive educator–child interaction is associated with good educator–parent communication levels [68,69]; an educator who invests in the child’s well-being at the day-care center is, in fact, more likely to construct an educational alliance with the parents as part of a joint responsibility pact in which the role and importance of the various adults involved in caring for and raising the child are mutually recognized.

In accordance with some studies [28], in order to foster the development of quality relationships with the parents too, it is essential to support precisely educators’ emotional skills development; for example, professional caregivers with good emotional competence find dialogue with families easier, because, in turn, they manage to talk about children’s emotions more easily [26]. The capacity to listen to and feel empathy for children as well as parents, to acknowledge and take due account of viewpoints that differ from one’s own, to constantly reflect on emotions and beliefs underlying their educational action, are all emotional competences that help safeguard and enrich the well-being of educators, children, and their families [68,70,71].

## 5. Study Limitations and Future Perspectives

The study has some limitations. The data were collected from a rather small sample of educators working in day-care centers in a large city in northern Italy, and therefore cannot be generalized to the entire Italian population.

The cross-sectional cut of the research, moreover, prevents an investigation of the trend of the considered variables and their reciprocal influences. Future longitudinal research could, for example, better investigate how the emotional socializer style of the educator can, over time, impact the child security at day-care center and his/her socio-emotional development.

Moreover, in this research, we used self-report instruments compiled by the educator; it would be useful, in future studies, to also employ other instruments, such as observation by an external observer to record the child security and his/her social competences or educator behaviors, thereby using more independent evaluators, limiting the bias deriving from social desirability and reinforcing the robustness of the results.

Finally, it would also be beneficial to take other child characteristics into account (for example temperament, quality of the relationship with the primary caregivers), along with those of the educator (for example, sensitivity), to analyze the relationship between the constructs in greater depth, including through analysis models that can better clarify the direct and indirect influences of the emotional competence of the educator on the well-being and adjustment of the child at the nursery.

## 6. Conclusions

The continuous training and professional competence of educators are key variables in promoting the quality of pre-school educational services in order to support the creation of a secure, stimulating environment, rich in opportunities for intensive verbal and social interaction and in experiences that are able to promote the ‘holistic’ development of children [72]. The results of our study strengthen the belief that it is essential, in training paths, to promote an approach that looks to a reflective professional practice focused on the awareness and development of emotional competence in educators, both in their personal aspects and relational dimensions, so as to construct not only adequate care relationships with children but also, from a contextual viewpoint [73], effective partnerships with parents, who support the ecological day-care–family transition.

## Figures and Tables

**Table 1:** Children’s characteristics.

Measures	Total Sample		Males		Females	
Age (Months)	M = 25.98	Range	M = 25.85	Range	M = 26.17	Range
	(SD = 5.41)	12–37	(SD = 5.33)	17–37	(SD = 5.52)	12–37
Age of entry into day care	Before 12	After 12	Before 12	After 12	Before 12	After 12
	months	months	months	months	months	months
	40.8%	59.6%	40.1%	59.9%	42%	58%
Attendance	Full time	Part time	Full time	Part time	Full time	Part time
	72.6%	27.4%	73.2%	26.8%	71.9%	28.1%

**Table 2:** CEESQ-Part 1: Descriptive statistics of the scales.

CEESQ-Scale Scores	Min	Max	M	SD	M/Item N	Skewness	Kurtosis
Coaching	17.00	35.00	26.93	3.32	3.85	−0.03	0.21
Dismissing	5.00	21.00	12.79	3.15	2.56	−0.22	−0.41
Self-efficacy as emotional socializer	13.00	30.00	20.36	2.77	3.43	0.57	0.25

**Table 3:** CEESQ-Part 1: Management of sadness and anger in children.

Item	Rarely or Never	A Bit	Quite	Often	Very Often
If children are sad, I don’t get involved unless it lasts too long or it is too intense	57.7%	22.3%	14.9%	4.7%	0.5%
Children’s sadness is an emotion worth exploring	0	2.7%	13.6%	48.4%	35.3%
When a child is angry, it’s an opportunity for getting close	0.9%	6.5%	33.2%	42.9%	16.6%
I am able to stay close to an angry child	0	1.8%	39.4%	49.3%	9.5%

**Table 4:** CEESQ-Part 2: Descriptive statistics of the scales.

CEESQ Second Part	Min	Max	M	SD	M/Item N	Skewness	Kurtosis
Emotional self-efficacy	22	44	31.39	3.94	3.14	0.83	0.73
Denial of emotions	4	16	8.09	2.27	2.02	0.41	0.30

**Table 5:** KIDI and Information Sources Questionnaire: descriptive statistics.

Questionnaire	Sub-Scales	Min	Max	M	SD	Skewness	Kurtosis
KIDI	Principles	−4	15	9.51	2.91	−0.87	1.70
	Parenting	2	14	10.23	2.42	−0.53	−10
Information sources	Teachers/educators	1	5	4.06	0.70	−0.63	0.97
	Articles/books	1	5	3.68	0.81	−0.07	−0.53
	Friends	1	5	2.89	0.89	0.11	−0.29
	Doctors	1	5	2.88	1.09	−0.04	−0.63
	Family	1	5	2.88	0.92	0.18	−0.17
	Partner	1	5	2.49	1.03	0.30	−0.42
	Mass media	1	5	2.00	0.86	1.34	2.33

**Table 6:** Attachment security and social skills at day-care—gender differences.

	Gender	N	M	SD	Skewness	Kurtosis
AQS t_(510)_ = −2.769 *p* < 0.01	M	281	111.58	16.35	−0.37	0.06
	F	231	115.42	14.63	−0.47	0.44
QPI—Global score t_(479.526)_ = −2.823; *p* < 0.01	M	283	59.33	9.63	−0.01	−0.37
	F	229	61.80	10.02	−0.12	−0.54
QPI—Difficulty in social participation t_(501.567)_ = 1.978; *p* < 0.05	M	298	9.36	2.70	0.77	0.55
	F	242	8.88	2.87	0.94	0.65
QPI—Positive social behaviors t_(537)_ = −3.858 *p* < 0.01	M	300	11.16	3.16	0.44	−0.45
	F	239	12.21	3.11	0.07	−0.64
QPI—Popularity t = n.s.	M	292	11.16	3.60	0.33	−0.52
	F	234	11.65	3.58	0.25	−0.47
QPI—Negative social behaviors t_(539)_ = 2.824 *p* < 0.01	M	300	13.76	2.61	0.13	0.28
	F	241	13.09	2.91	0.68	0.64

**Table 7:** Correlations between AQS and QPI for males and females.

Variables	Correlations											
	Males						Females					
	1	2	3	4	5	6	1	2	3	4	5	6
1. AQS	1	0.53 **	−0.39 **	0.51 **	0.33 **	−49 **	1	0.57 **	−0.48 **	0.43 **	0.48 **	−0.44 **
2. QPI—Global score		1	−78 **	0.83 **	0.82 **	−73 **		1	−0.79 **	0.83 **	0.84 **	−0.78 **
3. QPI—Difficulty in social participation			1	−52 **	−0.52 **	0.46 **			1	−0.53 **	−0.52 **	0.52 **
4. QPI—Positive social behaviors				1	0.56 **	−0.53 **				1	0.63 **	−0.51 **
5. QPI—Popularity					1	−0.41 **					1	−0.52 **
6. QPI—Negative social behaviors						1						1

** Correlation is significant at the 0.01 level (2-tailed).

**Table 8:** Correlations between children’s ages, AQS and QPI.

Item	Children’s Age	
	Males	Females
AQS	n.s.	n.s.
QPI—Global score	r = 0.32 ***	r = 0.34 ***
QPI—Difficulty in social participation	r = −0.20 ***	r = −0.28 ***
QPI—Positive social behaviors	r = 0.23 ***	r = 0.24 ***
QPI—Popularity	r = 0.38 ***	r = 0.28 ***
QPI—Negative social behaviors	r = −0.15 **	r = −0.31 ***

** *p* < 0.01; *** *p* < 0.001.

**Table 9:** Regression analyses on social skills and attachment security.

	Social Skills			Attachment Security		
	**F**	**R^2^**	**β**	**F**	**R^2^**	**β**
**Males**	4.79 **_(3, 249)_	0.05		4.37 **_(3, 247)_	0.05	
Educator’s coaching style			0.19 **			0.20 **
Educator’s dismissing style			−0.14 **			n.s
Educator’s self-efficacy as emotional socializer			n.s.			n.s
	**F**	**R^2^**	**β**	**F**	**R^2^**	**β**
**Females**	1.37_(3, 205)_	0.2		3.40 **_(3, 204)_	0.04	
Educator’s coaching style			n.s			0.22 **
Educator’s dismissing style			n.s			n.s.
Educator’s self-efficacy as emotional socializer			n.s.			n.s

** *p* < 0.01.

**Table 10:** Educator’s emotional socialization style: correlation analyses.

Variables	Correlations										
	1	2	3	4	5	6	7	8	9	10	11
1. CEESQ Coaching	1	0.03	0.54 **	0.47 **	−0.02	−0.09	−0.10	0.03	0.15 *	0.13	0.36 **
2. CEESQ Dismissing		1	0.15 *	0.08	0.18 *	−0.03	0.04	−0.16 *	−0.27 **	−0.11	0.08
3. CEESQ Emotional socializer self-efficacy			1	0.64 **	−0.22 **	−0.10	−0.08	−0.07	0.02	0.14	0.26 **
4. CEESQ Personal emotional self-efficacy				1	−0.32 **	−0.04	−0.07	0.01	0.02	0.10	0.25 **
5. CEESQ Denial of emotions					1	0.08	0.06	−0.07	−0.02	0.07	−0.06
6. Age						1	0.84	−0.13	0.10	−0.39	−0.13
7. Years of service							1	−0.15	0.07	−0.44	−0.14
8. KIDI Principles								1	0.30 **	0.11	−0.01
9. KIDI Parenting									1	0.03	−0.03
10. Level of training										1	0.06
11. QRS-F											1

* Correlation is significant at the 0.05 level (2-tailed). ** Correlation is significant at the 0.01 level (2-tailed).

## References

[1] Bowlby J. (1969). Attachment and Loss. Vol. 1. Attachment.

[2] Bowlby J. (1988). A Secure Base.

[3] Howes C., Cassidy J., Shaver P.R. (1999). Attachment relationships in the context of multiple caregivers. Handbook of Attachment.

[4] Cassibba R., Van Ijzendoorn M.H., D’Odorico L. (2000). Attachment and play in child care centres: Reliability and validity of the attachment Q-sort for mothers and professional caregivers in Italy. Int. J. Behav. Dev..

[5] Sylva K., Melhuish E., Sammons P., Siraj-Blatchford I., Taggart B. (2010). Early Childhood Matters. Evidence from the Effective Pre-School and Primary Education Project.

[6] Varin D. (2007). L’esperienza precoce ed estesa di asili nido: Fattori di facilitazione per lo sviluppo e aspetti di rischio. Psicol. Clin. dello Svilupp..

[7] Watamura S.E., Kryzer E.M., Robertson S.S. (2009). Cortisol patterns at home and child care. Afternoon differences and evening recovery in children attending very high quality full-day center-based child care. J. Appl. Dev. Psychol..

[8] NICHD Early Child Care Research Network (2002). Early child care and children’s development prior to school entry: Results from the NICHD study of early child care. Am. Educ. Res. J..

[9] Vandenbroeck M., Lenaerts K., Beblavy M. (2018). Benefits of Early Childhood Education and Care and the Conditions for Obtaining Them.

[10] Howes C., Phillips D.A., Whitebook M. (1992). Thresholds of quality: Implications for the social development of children in center-based child care. Child. Dev..

[11] Burchinal M.R., Peisner-Feinberg E., Pianta R., Howes C. (2002). Development of academic skills from preschool through second grade: Family and classroom predictors of developmental trajectories. J. Sch. Psychol..

[12] NICHD Early Child Care Research Network (1996). Characteristics of infant childcare; factors contributing to positive caregiving. Early Child. Res. Q..

[13] Phillipsen L.C., Burchinal M.R., Howes C., Cryer D. (1997). The prediction of process quality from structural features of child care. Early Child. Res. Q..

[14] Booth C.L., Kelly J.F., Spieker S.J., Zuckerman T.G. (2003). Toddlers’ Attachment Security to Child-Care Providers: The Safe and Secure Scale. Early Educ. Dev..

[15] Elfer P., Goldschmied E., Selleck D. (2003). Key Persons in the Nursery: Building Relationships for Quality Provision.

[16] Lally J.R. (1995). The impact of child care policies and practices on infant/toddler identity formation. Young Child..

[17] Shonkoff J.P., Phillips D.A. (2000). From Neurones to Neighbourhoods: The Science of Early Childhood Development.

[18] Whitington V., Ward C., Berk L. (1999). Intersubjectivity in Caregiver–Child Communication. Landscapes of Development: An Anthology of Readings.

[19] Wittmer D.S., Petersen S.H. (2005). Infant and Toddler Development and Responsive Program. Planning: A Relationship-Based Approach.

[20] Mata López C., Santelices Álvarez M.P., Vergés Gómez A. (2020). Do educators matter? Associations between caregivers’ mentalization and preschoolers’ attachment, social emotional development and theory of mind. Early Child. Dev. Care.

[21] Gottman J.M., Katz L.F., Hooven C. (1996). Parental meta-emotion philosophy and the emotional life of families: Theoretical models and preliminary data. J. Fam. Psychol..

[22] Gottman J., De Claire J. (1997). Raising an Emotionally Intelligent Child.

[23] Gottman J.M., Katz L.F., Hooven C. (1997). Meta-Emotion: How Families Communicate Emotionally.

[24] Cassibba R. (2003). Attaccamenti Multipli.

[25] Ciucci E., Baroncelli A., Toselli M. (2015). Meta-emotion philosophy in early childhood teachers: Psychometric properties of the Crèche Educator Emotional Styles Questionnaire. Early Child. Res. Q..

[26] Ciucci E., Baroncelli A., Toselli M., Denham S.A. (2018). Personal and professional emotional characteristics of early childhood teachers and their proneness to communicate with parents and colleagues about children’s emotions. Child. Youth Care Forum..

[27] Denham S.A., Bassett H.H., Zinsser K. (2012). Early childhood teachers as socializers of young children’s emotional competence. Early Child. Educ. J..

[28] Ornaghi V., Agliati A., Pepe A., Gabola P. (2020). Patterns of Association between Early Childhood Teachers’ Emotion Socialization Styles, Emotion Beliefs and Mind-Mindedness. Early Educ. Dev..

[29] Ahn H.J. (2005). Teachers’ discussions of emotion in child care centers. Early Child. Educ. J..

[30] Meyer D.K., Turner J.C., Schutz P.A., Pekrun R. (2007). Scaffolding emotions in classrooms. Emotion in Education.

[31] Schutz P.A., Zembylas M., Schutz P.A., Zembylas M. (2009). Introduction to advances in teacher emotion research: The impact on teachers’ lives. Advances in Teacher Emotion Research: The Impact on Teachers’ Lives.

[32] Yin H., Lee J.C. (2012). Be passionate, but be rational as well: Emotional rules for Chinese teachers’ work. Teach. Teach. Educ..

[33] Huntsman L. (2008). Determinants of Quality in Child Care: A Review of the Research Evidence.

[34] NICHD Early Child Care Research Network (2000). Characteristics and quality of child care for toddlers and preschoolers. Appl. Dev. Sci..

[35] Penrose A., Perry C., Ball I. (2007). Emotional intelligence and teacher self efficacy: The contribution of teacher status and length of experience. Issues Educ. Res..

[36] Hollingshead A.A. (1975). Four-Factor Index of Social Status.

[37] Scarzello D., Arace A., Prino L.E. (2016). Parental practices of Italian mothers and fathers during early infancy: The role of knowledge about parenting and child development. Infant. Behav. Dev..

[38] MacPhee D. (1981). Manual for the Knowledge of Infant Development Inventory.

[39] Bornstein M.H., Cote L.R., Haynes O.M., Hahn C.S., Park Y. (2010). Parenting knowledge: Experiential and sociodemographic factors in European American mothers of young children. Dev. Psychol..

[40] D’Odorico L., Cassibba R., Buono S. (2000). Le interazioni tra pari all’asilo nido: Metodi di valutazione e variabili rilevanti. Età Evol..

[41] Tallandini M.A., Morsan V. (2006). Competenza sociale e comprensione linguistica in età prescolare e scolare. Età Evol..

[42] Longobardi E., Spataro P., Frigerio A., Rescorla L. (2016). Gender differences in the relationship between language and social competence in preschool children. Infant. Behav. Dev..

[43] Waters E., Deane K.E. (1985). Defining and assessing individual differences in attachment relationships: Q-methodology and the organization of behavior in infancy and early childhood. Monogr. Soc. Res. Child. Dev..

[44] Cassibba R., D’Odorico L. (2000). La Valutazione Dell’attaccamento Nella PRIMA INFAnzia. L’adattamento Italiano Dell’attachment Q-Sort (AQS) Di Everett Waters.

[45] Marcuccio M., Zanelli P. (2013). Strumento Per lo Sviluppo Di Processi Riflessivi **e** Indagini Valutative Nei Nidi Da Parte Dei Gruppi Di Lavoro Educativi (SPRING).

[46] Arace A., Scarzello D., Zonca P., Agostini P. (2021). Early child care experiences and individual differences: The role of gender and temperament in social skills and problem behaviours in Italian toddlers. Early Child. Dev. Care.

[47] De Schipper J.C., Tavecchio L.W.C., Van IJzendoorn M.H. (2008). Children’s Attachment Relationships with Day Care Caregivers: Associations with Positive Caregiving and the Child’s Temperament. Soc. Dev..

[48] Degotardi S., Pearson E. (2009). Relationship Theory in the Nursery: Attachment and beyond. Contemp. Issues Early Child..

[49] Howes C., Smith E.W. (1995). Relations among child care quality, teacher behavior, children’s play activities, emotional security, and cognitive activity in child care. Early Child. Res. Q..

[50] Mitchell-Copeland J., Denham S.A., DeMulder E.K. (1997). Q-sort assessment of child–teacher attachment relationships and social competence in the preschool. Early Educ. Dev..

[51] Bowlby R. (2007). Babies and toddlers in non-parental daycare can avoid stress and anxiety if they develop a lasting secondary attachment bond with one carer who is consistently accessible to them. Attach. Hum. Dev..

[52] Commodari E. (2013). Preschool teacher attachment, school readiness and risk of learning difficulties. Early Child. Res. Q..

[53] Crockenberg S.C. (2003). Rescuing the baby from the bathwater: How gender and temperament (may) influence how child care affects child development. Child. Dev..

[54] Meland A.T., Kaltvedt E.H., Reikerås E. (2016). Toddlers master everyday activities in kindergarten: A gender perspective. Early Child. Educ. J..

[55] Howes C., Olenick M. (1986). Family and child care influences on toddler’s compliance. Child. Dev..

[56] Votruba-Drzal E., Coley R.L., Maldonado-Carreño C., Li-Grining C.P., Chase-Lansdale P.L. (2010). Child care and the development of behavior problems among economically disadvantaged children in middle childhood. Child. Dev..

[57] Bergin C., Bergin D. (2009). Attachment in the classroom. Educ. Psychol. Rev..

[58] Birch S.H., Ladd G.W. (1997). The teacher-child relationship and children’s early school adjustment. J. Sch. Psychol..

[59] Pianta R.C. (1999). Enhancing Relationships between Children and Teachers.

[60] Brody L.R., Hall J.A., Lewis M., Haviland J.M. (2000). Gender, emotion, and expression. Handbook of Emotions.

[61] Else-Quest N.M., Hyde J.S., Goldsmith H.H., Van Hulle C.A. (2006). Gender differences in temperament: A meta-analysis. Psychol. Bull..

[62] Maccoby E.E. (1998). The Two Sexes: Growing up Apart, Coming Together.

[63] Weinberg M.K., Tronick E.Z., Cohn J.F., Olson K.L. (1999). Gender differences in emotional expressivity and self-regulation during early infancy. Dev. Psychol..

[64] Chaplin T.M., Cole P.M., Zahn-Waxler C. (2005). Parental socialization of emotion expression: Gender differences and relations to child adjustment. Emotion.

[65] Dunn J., Bretherton I., Munn P. (1987). Conversations about feeling states between mothers and their young children. Dev. Psychol..

[66] Meece J.L., Painter J., Schunk D.H., Zimmerman B.J. (2008). Gender, self-regulation, and motivation. Motivation and Self-Regulated Learning: Theory, Research, and Applications.

[67] Zambrana I.M., Ystrom E., Pons F. (2012). Impact of gender, maternal education, and birth order on the development of language comprehension: A longitudinal study from 18 to 36 months of age. J. Dev. Behav. Pediatr..

[68] Capperucci D., Ciucci E., Baroncelli A. (2018). Relazione scuola-famiglia: Alleanza e corresponsabilità educativa. Riv. Ital. Educ. Fam..

[69] Owen M.T., Ware A.M., Barfoot B. (2000). Caregiver-Mother Partnership Behavior and the Quality of Caregiver-Child and Mother-Child Interactions. Early Child. Res. Q..

[70] Wharton A.S. (2009). The sociology of emotional labor. Annu. Rev. Sociol..

[71] Zembylas M. (2005). Beyond teacher cognition and teacher beliefs: The value of the ethnography of emotions in teaching. Int. J. Qual. Stud. Educ..

[72] (2014). Eurydice. Early Childhood Education and Care.

[73] Bronfenbrenner U. (1979). The Ecology of Human Development.

